# Cardiovascular Safety Landscape of ADT in Prostate Cancer Treatment Based on Real‐World Analysis

**DOI:** 10.1002/cam4.71487

**Published:** 2025-12-21

**Authors:** Wei Wang, Zirui Dong, Baoan Hong, Xin Guan, Yuxuan Wang, Zhipeng Sun, Qi Miao, Ning Zhang

**Affiliations:** ^1^ Department of Urology Beijing Anzhen Hospital, Capital Medical University Beijing P. R. China; ^2^ Department of Urology Union Hospital, Tongji Medical College, Huazhong University of Science and Technology Wuhan Hubei P. R. China; ^3^ Department of Nuclear Medicine First Hospital of Shanxi Medical University, Shanxi Medical University Taiyuan Shanxi P. R. China

**Keywords:** ADT, cardiovascular toxicity, prostate cancer

## Abstract

**Background:**

Prostate cancer is among the most prevalent malignancies worldwide, and cardiovascular disease (CVD) is a major non‐cancer cause of death in affected patients. Androgen deprivation therapy (ADT), a mainstay treatment, has raised concerns about cardiotoxicity, yet the CVD risks of individual ADT agents remain unclear.

**Objectives:**

To assess cardiovascular adverse events (AEs) associated with specific ADT drugs using data from the U.S. FDA Adverse Event Reporting System (FAERS).

**Methods:**

AE reports related to ADT drugs were extracted from FAERS (Q1 2004–Q3 2024). Disproportionality analyses—including Reporting Odds Ratio (ROR) and Proportional Reporting Ratio (PRR)—were conducted to identify significant cardiovascular safety signals.

**Results:**

Different ADT agents exhibited distinct cardiovascular AE profiles. Some drugs were linked to a broader range of CVD‐related AEs, while others had more limited associations.

**Conclusions:**

ADT agents demonstrate heterogeneous cardiotoxicity profiles. These findings emphasize the need for individualized treatment strategies, particularly in patients with pre‐existing CVD risks, and may aid clinicians in balancing cancer control with cardiovascular safety.

## 1 Introduction

Prostate cancer remains one of the most prevalent malignancies worldwide and continues to impose a substantial public health burden. In 2025, it is projected that approximately 313,780 new cases will be diagnosed, representing about 30% of all newly reported cancers in men, with an estimated 35,770 deaths (11%) attributed to the disease [1]. Advances in the early detection and treatment of prostate cancer have substantially improved patient survival. However, with increased longevity, the management of comorbidities—particularly cardiovascular disease (CVD)—has become a critical aspect of care. Cardiovascular complications now represent one of the leading non‐cancer causes of mortality among prostate cancer patients. An expanding body of evidence underscores the complex relationship between prostate cancer therapies and cardiovascular risk, with certain treatments either aggravating preexisting CVD or conferring additional cardiovascular complications [2, 3, 4]. The complex interplay between prostate cancer and CVD highlights the necessity of comprehensive management strategies aimed at mitigating cardiovascular risk while simultaneously optimizing oncologic outcomes. A thorough understanding of how prostate cancer treatments affect cardiovascular health is essential for improving long‐term survival and enhancing the quality of life of affected patients.

Androgen deprivation therapy (ADT)—also referred to as androgen ablation or androgen suppression—is a widely used hormonal treatment for prostate cancer [5]. As a standard therapeutic approach, ADT is widely administered in patients with primary prostate cancer, oligometastatic disease, and metastatic castration‐sensitive prostate cancer [6, 7]. However, the potential association between ADT and increased cardiovascular mortality in men with prostate cancer remains a subject of ongoing debate. Furthermore, whether individual ADT agents confer distinct cardiovascular risks has not yet been fully clarified [8, 9, 10]. Recently, increasing concerns regarding the cardiovascular adverse effects of prostate cancer treatments have underscored the need for a more personalized therapeutic approach, highlighting the importance of precision medicine in clinical decision‐making [11]. Furthermore, high‐quality systematic reviews and network meta‐analyses have emphasized the cardiotoxic risks associated with various prostate cancer therapies, underscoring the need for further research to optimize patient outcomes [12].

The FDA Adverse Event Reporting System (FAERS) is a publicly accessible database maintained by the U.S. Food and Drug Administration (FDA) to monitor drug safety and detect potential adverse drug reactions (ADRs). It compiles spontaneous reports of adverse events, medication errors, and product quality issues submitted by healthcare professionals, consumers, and manufacturers. FAERS plays a pivotal role in post‐marketing surveillance by providing real‐world evidence on drug safety, facilitating risk assessment, and informing regulatory decision‐making. By leveraging large‐scale pharmacovigilance data, FAERS has been extensively used to identify potential drug‐associated risks, including cardiovascular complications related to prostate cancer therapies [13].

To further elucidate the cardiotoxicity associated with prostate cancer treatments, we conducted an investigation from an alternative perspective. By mining the FAERS database, we identified cardiovascular adverse events associated with different FDA‐approved ADT agents using two statistical models. This study aims to provide a comprehensive and in‐depth characterization of CVD‐related adverse events linked to ADT, offering a valuable reference for clinical decision‐making regarding the selection of ADT agents in patients with preexisting CVD or at high cardiovascular risk.

## 2 Methods

### 2.1 Data Sources and Extraction

The FAERS database is a spontaneous reporting system that compiles adverse event (AE) reports, medication errors, and product quality complaints associated with AEs from healthcare providers, consumers, and pharmaceutical manufacturers. As a vital resource for post‐marketing drug and biologic safety surveillance, FAERS has accumulated over 21 million AE reports. The database is structured into seven distinct data files: drug details (DRUG), patient outcomes (OUTC), sources of reports (RPSR), therapy initiation and discontinuation dates (THER), drug indication data (INDI), and demographic and administrative records (DEMO). These datasets are interconnected through unique identifiers, such as PRIMARYID, which is assigned to each FAERS report to maintain data integrity and facilitate comprehensive analysis.

In this study, we identified adverse event (AE) reports related to androgen deprivation therapy (ADT) drugs—including abiraterone, apalutamide, bicalutamide, darolutamide, degarelix, dexamethasone, enzalutamide, flutamide, goserelin, hydrocortisone, ketoconazole, leuprolide, nilutamide, prednisone, and triptorelin—by searching both their brand and generic names. All reported AEs were categorized using the Medical Dictionary for Regulatory Activities (MedDRA 27.1) and mapped to preferred terms (PTs) within the system organ class (SOC) framework. The hierarchical structure of MedDRA terminology progresses from lowest level terms (LLT) to PT, high‐level terms (HLT), high‐level group terms (HLGT), and SOC.

FAERS reports from Q1 2004 to Q3 2024 were included in our study. To enhance data reliability and minimize redundancy, we performed rigorous data preprocessing. Duplicate reports were removed following FDA guidelines, ensuring that only the most recent version of each case was retained. Additionally, only cases in which the ADT drug was designated as the “primary suspect” were included, while reports where the drug was labeled as “concomitant” or “interacting” were excluded. Given that FAERS is a publicly accessible database, this study did not require institutional review board approval or patient consent.

### 2.2 Data Analysis

Disproportionality analyses are a cornerstone of pharmacovigilance for identifying potential drug safety signals, yet no universally accepted standard for signal detection exists. To enhance the reliability of drug‐AE association assessments, this study utilized two established disproportionality analysis methods: the reporting odds ratio (ROR) and the Proportional Reporting Ratio (PRR), along with their respective 95% confidence intervals (CIs). Table 1 outlines the formulas and signal detection thresholds used in these analyses. A signal was considered statistically significant under the following conditions: (1) the lower bound of the 95% CI for ROR exceeded 1, with at least three reports; and (2) PRR ≥ 2, *χ* ^2^ ≥ 4, *N* ≥ 3; If both criteria were met, the AE was classified as having a significant safety signal. In general, higher values from these algorithms indicate stronger signals, reflecting a more pronounced association between the drug and the AE.

**TABLE 1:** Two major algorithms used for signal detection.

Algorithms	Equation	Criteria
ROR	$\mathrm{ROR}=\frac{a/c}{b/d}$ $95\%\mathrm{CI}=e^{\ln\left(\mathrm{ROR}\right)\pm 1.96\sqrt{\frac{1}{a}+\frac{1}{b}+\frac{1}{c}+\frac{1}{d}}}$	Lower limit of 95% CI > 1, *N* ≥ 3
PRR	$\begin{array}{l} \mathrm{PRR}=\left[a\left(c+d\right)\right]/\left[c\left(a+b\right)\right]\\ x^{2}=\frac{\left(a+b+c+d\right){\left(\mathrm{ad}‐\mathrm{bc}\right)}^{2}}{\left(a+b\right)\left(c+d\right)\left(a+c\right)\left(b+d\right)} \end{array}$	PRR ≥ 2, χ^2^ ≥ 4, *N* ≥ 3

Abbreviations: 95% CI, 95% confidence interval; a, number of reports containing both the target drug and target adverse drug reaction; b, number of reports containing other adverse drug reactions of the target drug; c, number of reports containing the target adverse drug reaction of other drugs; d, number of reports containing other drugs and other adverse drug reactions; *N*, the number of reports; PRR, Proportional Reporting Ratio; ROR, reporting odds ratio.

### 2.3 Statistical Analysis

All statistical analyses and data processing were performed using R software (version 4.4.1) and Microsoft Excel 2019. To assess differences in the occurrence of CVD‐related AEs among patients receiving ADT therapy, the chi‐square test was utilized. This study adheres strictly to the READUS‐PV guidelines, which were designed to establish standardized methodologies for signal detection within the FAERS database.

## 3 Results

### 3.1 Discriptional Analysis

**FIGURE 1:**
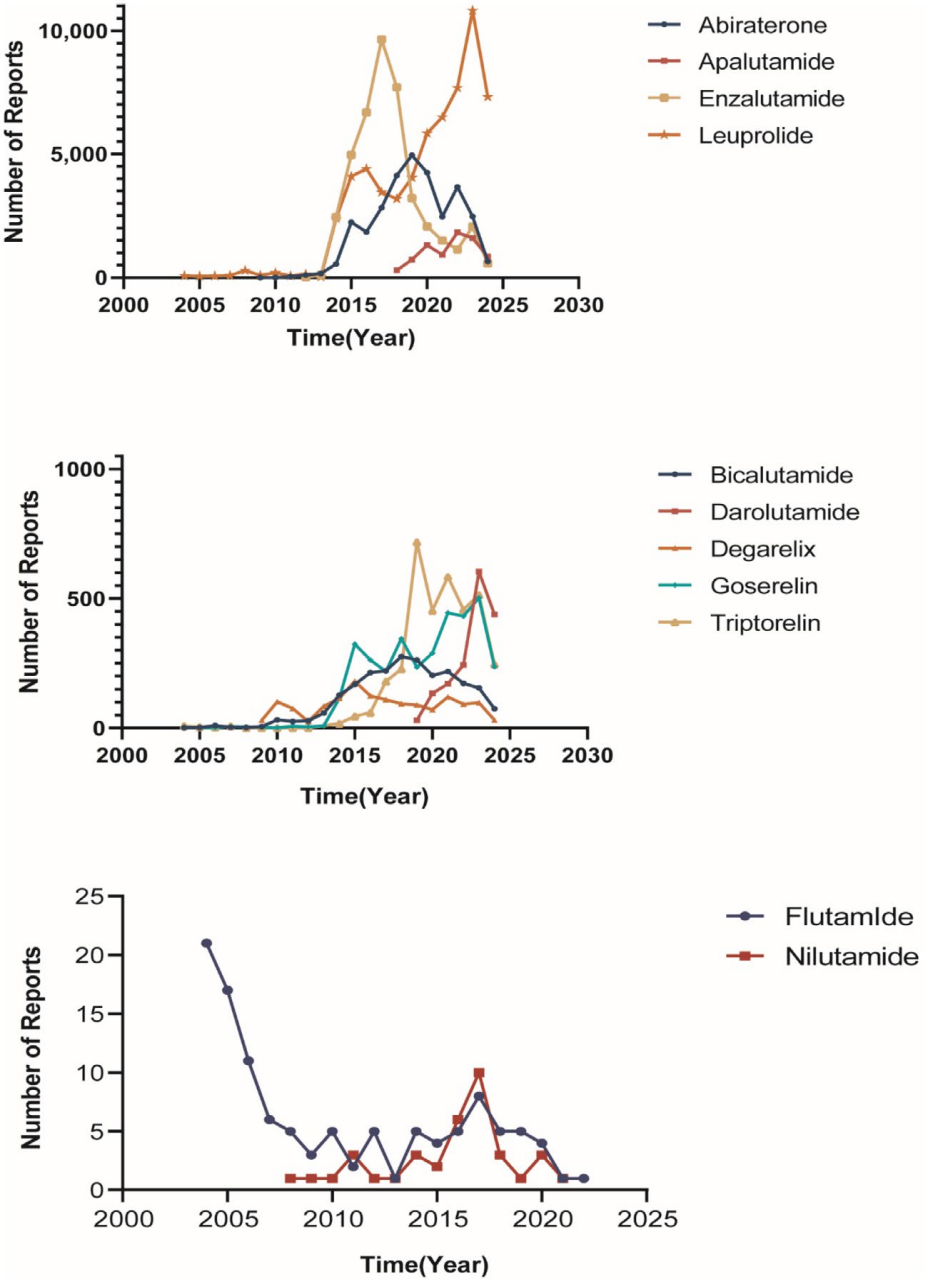
Time trend of cardiovascular‐related diseases associated with ADT.

From Q1 2004 to Q3 2024, a total of 153,354 adverse event reports related to cardiovascular diseases (CVDs) associated with ADT drugs were recorded. The number of events for each agent was as follows: abiraterone (30,366), apalutamide (7566), bicalutamide (2261), darolutamide (1621), degarelix (1445), enzalutamide (42,145), flutamide (114), goserelin (3449), leuprolide (60,817), nilutamide (37), and triptorelin (3533). Figure 1 depicts the temporal trends of CVD‐related adverse events associated with ADT therapy. Notably, AE reports for enzalutamide peaked in 2017 before declining, whereas reports for leuprolide exhibited a continuous upward trend, reaching their highest level in 2023. Table 2 summarizes the clinical characteristics of patients experiencing CVDs associated with ADT agents. For instance, in patients treated with abiraterone, CVDs were most frequently observed in individuals aged 65–85 years, accounting for 39.21% of cases. Regarding clinical outcomes, mortality was considerable, occurring in 21.59% of cases, while hospitalization—either initial or prolonged—was reported in 19.95% of cases.

**TABLE 2:** Clinical characteristics of cardiovascular adverse events reported on ADT drugs.

	Abiraterone	Apalutamide	Bicalutamide	Darolutamide	Degarelix	Enzalutamide	Flutamide	Goserelin	Leuprolide	Nilutamide	Triptorelin
Age (years)											
< 18	16	26	15	5	3	33	2	2	1557	0	662
18~64.9	2547	399	314	179	78	3062	16	230	8550	4	246
65~85	11,842	2949	1227	737	447	16,811	58	311	18,232	22	665
> 85	2296	605	165	153	74	4066	4	84	3402	7	121
Missing	13,665	3587	540	547	843	18,173	34	2822	53,923	4	1839
Weight (kg)											
< 50	123	39	13	7	28	157	1	21	531	0	30
50~100	3879	1021	708	61	229	1099	19	163	5633	15	182
> 100	739	160	149	9	15	329	5	22	730	2	21
Missing	25,625	6346	1391	1544	1173	40,560	89	3243	53,923	20	3300
Outcome											
Congenital anomaly	1	1	NA	NA	NA	3	1	1	17	NA	2
Death	6521	1068	265	160	238	9030	29	1249	10,091	5	328
Disability	79	26	56	3	8	102	1	58	425	1	46
Hospitalization	6027	1344	725	297	595	5795	43	443	7066	6	508
Life‐threatening	383	108	146	11	39	289	7	57	428	2	33
Required intervention to prevent	22	10	2	2	NA	5	2	1	16	NA	1
Other	5576	1820	848	650	353	9709	27	1129	10,300	16	761
Missing	11,757	3189	219	498	212	17,212	4	511	32,474	7	1854
Total	30,366	7566	2261	1621	1445	42,145	114	3449	60,817	37	3533

### 3.2 Adverse Events Based on Drug Type

**FIGURE 2:**
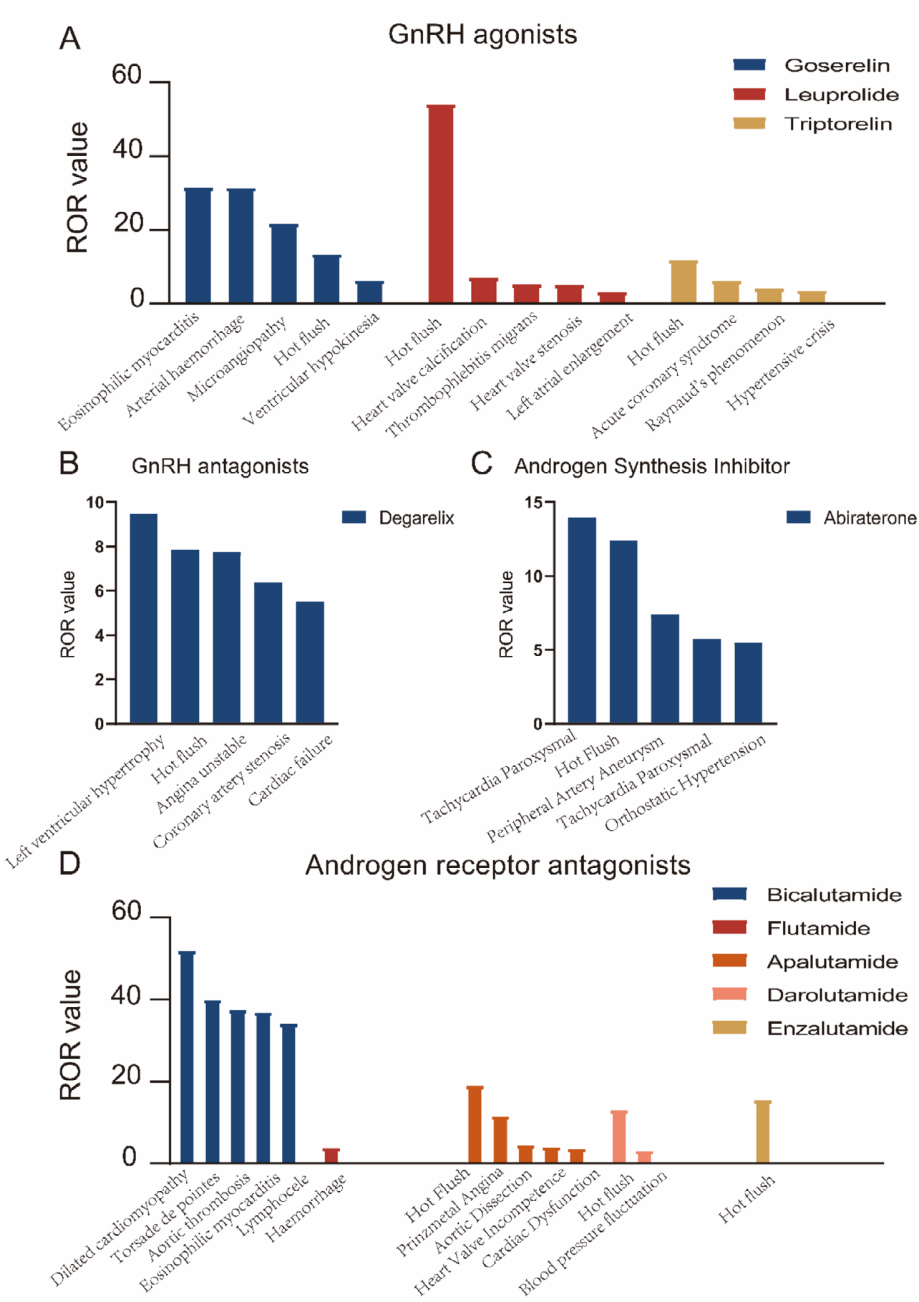
Top 5 cardiovascular adverse effects based on drug type (ROR value).

Next, we analyzed the cardiovascular adverse events associated with each ADT agent, and the results are presented in Figure 2 and Figure S1.

#### 3.2.1 GnRH Agonists (Figure 2A and Figure S1A)

Cardiovascular adverse events (AEs) associated with the GnRH agonists goserelin, leuprolide, and triptorelin are detailed in Tables S7–S9. For goserelin, five cardiovascular AEs were identified, of which only arterial hemorrhage was not listed in the FDA‐approved prescribing information. In the case of leuprolide, the only AE among those identified that was included in the FDA label was hot flush. Notably, triptorelin was the only GnRH agonist in our study for which all detected cardiovascular AEs were already documented in the FDA prescribing information.

#### 3.2.2 GnRH Antagonists (Figure 2B and Figure S1B)

Cardiovascular AEs associated with degarelix are listed in Table S5. Among these, only hot flush, heart failure, cardiomegaly, coronary artery stenosis, and acute heart failure were included in FDA‐approved labeling.

#### 3.2.3 Androgen Receptor Antagonists (Figure 2D and Figure S1D)

##### 3.2.3.1 First Generation

Among first‐generation antiandrogens, bicalutamide exhibited the broadest spectrum of cardiovascular toxicity‐related AEs, with a total of 40 distinct events identified (Table S3). These included clinically significant conditions such as hot flush, cardiac failure, torsades de pointes, hypertensive crises, acute coronary syndrome, cardiomegaly, cardiac dysfunction, Raynaud's phenomenon, mitral valve regurgitation, supraventricular tachycardia, eosinophilic myocarditis, microangiopathy, and impaired ventricular motion, many of which are explicitly listed in the FDA‐approved prescribing information. However, a substantial proportion of the AEs identified in our analysis were not included in the FDA labeling, suggesting potential under‐recognition in formal safety documentation. In contrast, flutamide was associated with only a single cardiovascular AE—hemorrhage (ROR [L, U]: 3.81 [1.22, 11.85]; PRR [L, U]: 3.79 [2.66, 4.92]). Interestingly, nilutamide did not demonstrate any statistically significant cardiovascular AEs in our analysis. Whether this absence reflects a true lack of cardiotoxicity or is due to inherent limitations of the FAERS system warrants further investigation using real‐world clinical data and prospective studies [14].

##### 3.2.3.2 Second Generation

Among the second‐generation antiandrogens, apalutamide was associated with 13 cardiovascular adverse events, as identified by both the ROR and PRR models (Table S2). Of these, only hot flush, hypertension, cardiac failure, hypertensive emergency, acute coronary syndrome, heart valve incompetence, aortic dissection, cardiac dysfunction, and supraventricular tachycardia were explicitly documented in the FDA‐approved prescribing information, while the remaining events were not officially labeled. Darolutamide was linked to two cardiovascular AEs—hot flush and blood pressure fluctuation. Notably, only hot flush was included in the FDA label, whereas blood pressure fluctuation, although detected in our statistical analysis, was not explicitly mentioned (Table S4). For enzalutamide, our analysis identified hot flush as the sole cardiovascular AE with a statistically significant association (ROR [L, U]: 15.52 [14.86, 16.21]; PRR [L, U]: 15.27 [15.22, 15.31]). This observation aligns with existing literature indicating that enzalutamide generally exhibits a milder cardiotoxicity profile compared to other ADT agents [15].

#### 3.2.4 Androgen Synthesis Inhibitor (Figure 2C and Figure S1C)

Based on the ROR and PRR models, a total of 17 cardiovascular adverse events were associated with abiraterone (Table S1). Notably, among these events, only hot flush, hypertension, cardiac failure, acute coronary syndrome, torsade de points, hypertensive crisis, acute cardiac failure, and aortic dissection were explicitly listed in the FDA‐approved prescribing information, whereas the remaining adverse events were not included.

### 3.3 Adverse Events Based on Disease Type

**FIGURE 3:**
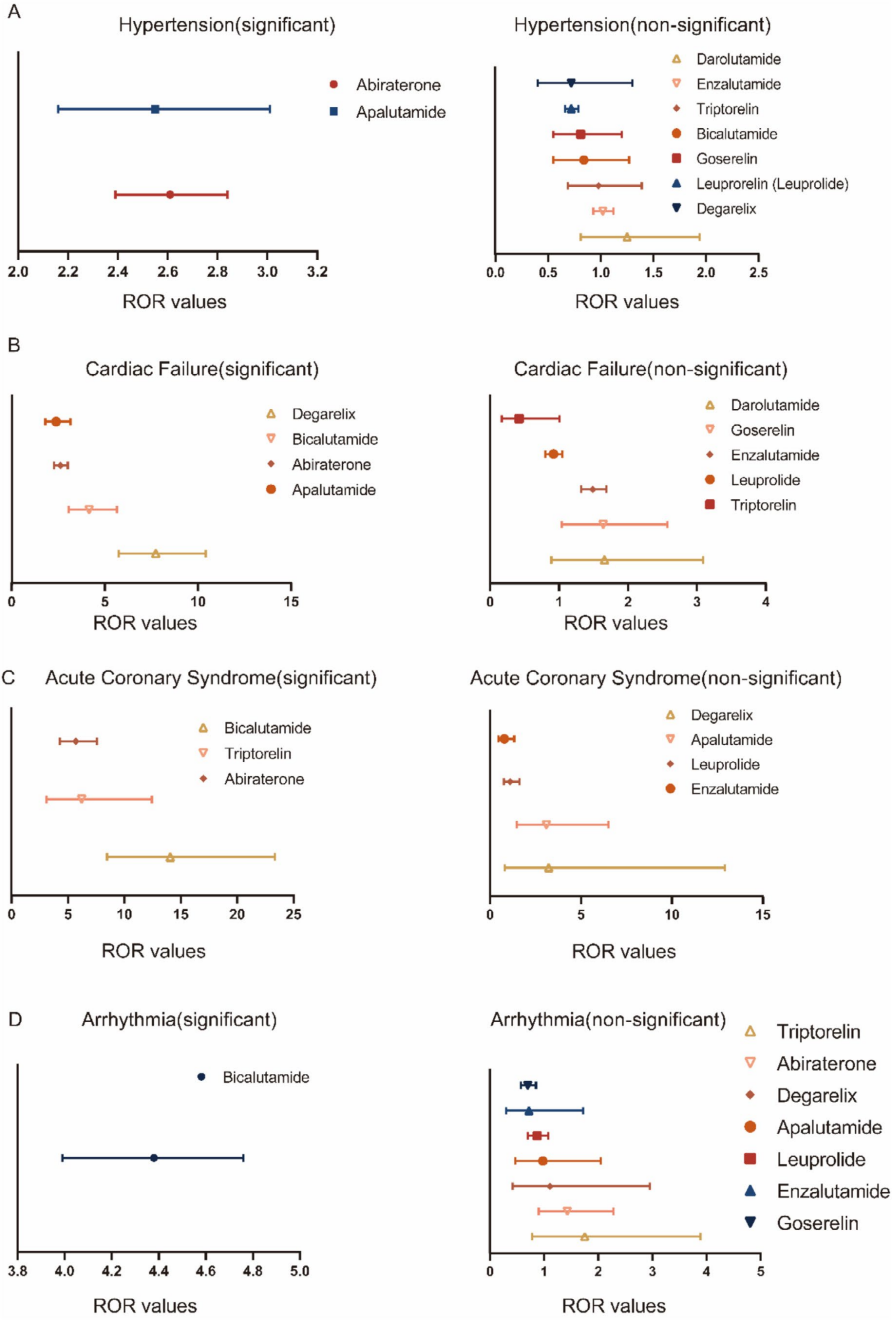
ADT drugs related adverse events based on disease type according to ROR value. (A) ADT drugs related to Hypertension ranked by ROR value. (B) ADT drugs related to Cardiac Failure ranked by ROR value. (C) ADT drugs related to Cardiac Failure ranked by ROR value. (D) ADT drugs related to Cardiac Failure ranked by ROR value.

To improve the clinical relevance of ADT drug selection, we focused on four of the most severe and prevalent cardiovascular adverse events. We analyzed the associations of these events with individual ADT agents and conducted a comparative assessment to evaluate their relative cardiovascular risk profiles (Figure 3).

#### 3.3.1 Hypertension(Figure S2A and Table S10)

Hypertension is the leading contributor to a global disease burden and a major cause of mortality worldwide [16]. The relationship between hypertension and prostate cancer therapy has recently garnered increasing recognition and clinical attention [17].

Among the nine ADT agents analyzed, abiraterone (ROR [L, U]: 2.61 [2.39, 2.84]; PRR [L, U]: 2.59 [2.51, 2.68]) and apalutamide (ROR [L, U]: 2.55 [2.16, 3.01]; PRR [L, U]: 2.54 [2.37, 2.70]) exhibited a strong association with hypertension‐related adverse events, as indicated by both ROR and PRR metrics.

It is important to note that our analysis was specifically based on FAERS data, in which the preferred term (PT) was labeled as “Hypertension.” Certain conditions closely related to hypertension may not have been captured due to variations in PT nomenclature. For further details, see Table S10. Interestingly, even when considering various subtypes of hypertension, abiraterone remained the ADT agent most strongly associated with hypertensive events.

#### 3.3.2 Cardiac Failure (Figure S2B)

The clinical relationship between heart failure (HF) and incident cancer remains a subject of ongoing debate [18].

In our analysis of nine ADT agents, degarelix (ROR [L, U]: 7.74 [5.75, 10.42]; PRR [L, U]: 7.67 [7.38, 7.97]), bicalutamide (ROR [L, U]: 4.16 [3.06, 5.65]; PRR [L, U]: 4.14 [3.83, 4.44]), abiraterone (ROR [L, U]: 2.63 [2.29, 3.01]; PRR [L, U]: 2.62 [2.48, 2.76]), and apalutamide (ROR [L, U]: 2.39 [1.81, 3.16]; PRR [L, U]: 2.39 [2.11, 2.67]) were identified as strongly associated with HF in prostate cancer patients, as indicated by both ROR and PRR analyses.

Given the inherent characteristics of the FAERS database, we further examined HF subtypes to provide more granular insights. Specifically, acute cardiac failure associated with degarelix yielded an ROR of 6.61 [2.13, 20.5] and a PRR of 6.6 [5.47, 7.74], while acute cardiac failure associated with abiraterone yielded an ROR of 2.1 [1.22, 3.62] and a PRR of 2.1 [1.55, 2.64]. Additionally, left ventricular failure was associated with bicalutamide (ROR: 9.14 [3.43, 24.39]; PRR: 9.14 [8.16, 10.12]). This supplementary analysis provides further insights into the potential relationships between these ADT agents and specific HF subtypes.

#### 3.3.3 Acute Coronary Syndrome (Figure S2C)

Despite substantial advancements in the diagnosis and management of acute coronary syndromes (ACS), cardiovascular disease remains the leading cause of death globally, with nearly half of these fatalities attributable to ischemic heart disease [19]. However, dedicated studies investigating the relationship between ACS and prostate cancer therapies remain limited. In our analysis of nine ADT agents, bicalutamide (ROR [L, U]: 14.06 [8.47, 23.34]; PRR [L, U]: 14.03 [13.53, 14.54]), triptorelin (ROR [L, U]: 6.22 [3.11, 12.44]; PRR [L, U]: 6.21 [5.52, 6.9]), and abiraterone (ROR [L, U]: 5.7 [4.29, 7.58]; PRR [L, U]: 5.7 [5.42, 5.98]) were identified as the agents most strongly associated with ACS, as indicated by both ROR and PRR analyses.

#### 3.3.4 Arrhythmia (Figure S2D)

Arrhythmias are increasingly recognized as a complication in cancer patients, posing unique management challenges [20]. Based on our statistical analysis, only bicalutamide was significantly associated with arrhythmia (ROR [L, U]: 4.39 [2.99, 6.45]; PRR [L, U]: 4.38 [3.99, 4.76]). Consistent with the observations in other cardiovascular events, arrhythmia encompasses a wide range of subtypes, resulting in complex scenarios with respect to Preferred Term (PT) nomenclature. To address this complexity, we have systematically organized and summarized the relevant data, which are presented in Table 3 for reference and further analysis.

**TABLE 3:** Cardiovascular adverse events related to arrhythmia.

Drug name	PT name	ROR [L, U]	PRR [L, U]
Abiraterone	Tachycardia paroxysmal	13.96 [5.2, 37.47]	13.96 [12.97, 14.95]
	Torsade de pointes	3.69 [2.55, 5.35]	3.69 [3.32, 4.06]
	Atrial fibrillation	2.6 [2.3, 2.95]	2.59 [2.47, 2.72]
Apalutamide	Supraventricular tachycardia	2.37[1.06, 5.28]	2.37 [1.57, 3.17]
Bicalutamide	Torsade de pointes	39.86 [28.95, 54.87]	39.66 [39.34, 39.98]
	Atrioventricular block first degree	10.62 [4.77, 23.66]	10.61 [9.81, 11.41]
	Long QT syndrome	10.34 [3.33, 32.11]	10.34 [9.21, 11.47]
	Atrioventricular block complete	9.85 [4.92, 19.72]	9.84 [9.15, 10.54]
	Ventricular arrhythmia	8.87 [3.33, 23.66]	8.87 [7.89, 9.85]
	Ventricular fibrillation	7.18 [3.86, 13.35]	7.17 [6.55, 7.79]
	Bundle branch block right	6.77 [2.54, 18.06]	6.77 [5.79, 7.75]
	Ventricular extrasystoles	6.07 [3.03, 12.15]	6.07 [5.37, 6.76]
	Atrial flutter	4.94 [2.06, 11.88]	4.94 [4.06, 5.82]
	Supraventricular tachycardia	3.34 [1.25,8.9]	3.34 [2.36, 4.32]
	Sinus bradycardia	3.34 [1.25, 8.91]	3.34[2.36, 4.32]
	Atrioventricular block	3.15 [1.02, 9.78]	3.15 [2.02, 4.28]
	Ventricular tachycardia	2.93 [1.32, 6.53]	2.93 [2.13, 3.73]

## 4 Discussion

The complex and clinically significant interplay between prostate cancer and cardiovascular comorbidities has long been discussed [21]. Nevertheless, these comorbidities are frequently underdiagnosed and inadequately managed according to established cardiovascular practice guidelines. Such gaps in care are particularly concerning in the context of androgen deprivation therapy (ADT), a cornerstone of pharmacologic treatment for prostate cancer. Although ADT effectively suppresses androgen levels to inhibit tumor progression, emerging evidence indicates that it may also elevate the risk of cardiovascular AEs [22]. Despite these concerns, the cardiovascular safety profiles of individual ADT agents remain insufficiently characterized, and these risks are often underappreciated in clinical decision‐making [11].

In this study, we conducted a systematic analysis of the FAERS database to evaluate cardiovascular AEs associated with ADT agents. Using two robust statistical models, we identified and summarized cardiovascular risks across different ADT therapies. Understanding which ADT agents carry a higher or lower risk of cardiovascular complications can help tailor treatment decisions to minimize adverse outcomes. Moreover, for patients already at an increased risk of developing CVD, our study underscores the importance of proactive cardiovascular monitoring during ADT therapy as mentioned by previous study [23]. Specifically, based on our statistical analysis, distinct differences in cardiovascular adverse events (CVD‐related AEs) were observed across different classes of ADT agents. Among GnRH agonists, triptorelin was associated with the lowest number of CVD‐related AEs. Within androgen receptor antagonists, nilutamide and enzalutamide showed the lowest number of CVD‐related AEs among the first‐ and second‐generation agents, respectively, whereas bicalutamide and apalutamide exhibited the highest number of CVD‐related AEs within the same categories. When focusing on major cardiovascular outcomes, abiraterone and apalutamide were most strongly associated with hypertension. Degarelix, bicalutamide, abiraterone, and apalutamide showed a higher association with cardiac failure. Bicalutamide, triptorelin, and abiraterone were more frequently linked to acute coronary syndrome. Additionally, bicalutamide, apalutamide, and abiraterone were strongly associated with arrhythmia. These findings may provide a useful reference for drug selection in clinical practice, particularly when managing patients at high risk of cardiovascular disease. Furthermore, an important aspect of our study is the identification of cardiovascular AEs associated with ADT agents that are not explicitly listed in FDA‐approved drug labeling. Our statistical analysis detected significant associations between certain ADT drugs and cardiovascular complications that lack formal recognition in regulatory documentation. This discrepancy highlights the need for increased clinical awareness.

Overall, our study underscores the importance of integrating cardiovascular risk assessment and management into prostate cancer treatment strategies. Clinicians should carefully balance the therapeutic benefits of ADT against potential cardiovascular risks, particularly in patients with preexisting cardiovascular disease (CVD) or those at elevated risk for developing cardiovascular complications. Future research should aim to validate these findings in prospective clinical studies, with a focus on refining risk stratification approaches and optimizing individualized treatment plans. By bridging the gap between oncology and cardiology, we hope that our work contributes to enhancing both the safety and efficacy of ADT in the management of prostate cancer.

Despite the strengths of this study, several limitations warrant consideration. The most notable limitation arises from the inherent characteristics of the FAERS database, which captures adverse events reported across all populations exposed to a given drug, rather than restricting observations to a specific disease context. Although most ADT agents are primarily prescribed for prostate cancer, a subset of patients may receive these medications for alternative indications [24]. Consequently, the observed cardiovascular toxicities may not be exclusively attributable to the interaction between ADT and prostate cancer–specific pathophysiology but may also reflect the broader cardiovascular effects of ADT across diverse patient populations. This distinction is critical, as the interplay between ADT, prostate cancer, and cardiovascular toxicity may differ from the cardiovascular risks encountered in patients receiving ADT for non‐prostate cancer indications.

## Author Contributions

Ning Zhang and Qi Miao conceived the present study. Wei Wang, Zirui Dong, Baoan Hong, and Xin Guan drafted the manuscript. Yuxuan Wang, Xuezhou Zhang, and Zhipeng Sun were significant contributors to the revision of the manuscript. All authors read and approved the final manuscript.

## Funding

This study was supported by the Beijing Hospitals Authority Youth Programme (QML20231105), Beijing Bethune Charitable Foundation (mnzl202003), Beijing Anzhen Hospital High Level Research Funding (2024AZC3001), Beijing Anzhen Hospital Science and Technology Development Funding (AZYZR202304).

## Disclosure

All collected data will be made available on request immediately after publication.

## Conflicts of Interest

The authors declare no conflicts of interest.

## Supporting information


**Figure S1:** Top 5 Cardiovascular adverse effects based on drug type (PRR value).
**Figure S2:**: ADT drugs related adverse events based on disease type according to PRR value. (A) ADT drugs related with Hypertension ranked by PRR value. (B) ADT drugs related with Cardiac Failure ranked by PRR value. (C) ADT drugs related with Cardiac Failure ranked by PRR value.
**Table S1:** Cardiovascular adverse events of Abiraterone.
**Table S2:** Cardiovascular adverse events of Apalutamide.
**Table S3:** Cardiovascular adverse events of Bicalutamide.
**Table S4:** Cardiovascular adverse events of Darolutamide.
**Table S5:** Cardiovascular adverse events of Degarelix.
**Table S6:** Cardiovascular adverse events of Degarelix.
**Table S7:** Cardiovascular adverse events of Goserelin.
**Table S8:** Cardiovascular adverse events of Leuprolide.
**Table S9:** Cardiovascular adverse events of Triptorelin.
**Table S10:** Cardiovascular Adverse Events related with hypertension.

## Data Availability

All the data can be retrieved from the FAERS Publish Dashboard (https://www.fda.gov/drugs/questions‐and‐answers‐fdas‐adverse‐event‐reporting‐system‐faers/fda‐adverse‐event‐reporting‐system‐faers‐public‐dashboard).
