# Supplementary material for: Cardiovascular Safety Landscape of ADT in Prostate Cancer Treatment Based on Real‐World Analysis
Source: Cancer Med. 2025 Dec 21;14(24):e71487. doi: 10.1002/cam4.71487 (PMC12719240; doi:10.1002/cam4.71487)
Supplement: Supplementary file 1 — Figure S1: Top 5 Cardiovascular adverse effects based on drug type (PRR value). Figure S2:: ADT drugs related adverse events based on disease type according to PRR value. (A) ADT drugs related with Hypertension ranked by PRR value. (B) ADT drugs related with Cardiac Failure ranked by PRR value. (C) ADT drugs related with Cardiac Failure ranked by PRR value. Table S1: Cardiovascular adverse events of Abiraterone. Table S2: Cardiovascular adverse events of Apalutamide. Table S3: Cardiovascular adverse events of Bicalutamide. Table S4: Cardiovascular adverse events of Darolutamide. Table S5: Cardiovascular adverse events of Degarelix. Table S6: Cardiovascular adverse events of Degarelix. Table S7: Cardiovascular adverse events of Goserelin. Table S8: Cardiovascular adverse events of Leuprolide. Table S9: Cardiovascular adverse events of Triptorelin. Table S10: Cardiovascular Adverse Events related with hypertension. [file CAM4-14-e71487-s001.docx]

**Supplementary Information:**

**Supplementary Figure 1: Top 5 Cardiovascular adverse effects based on drug type(PRR value)**

**
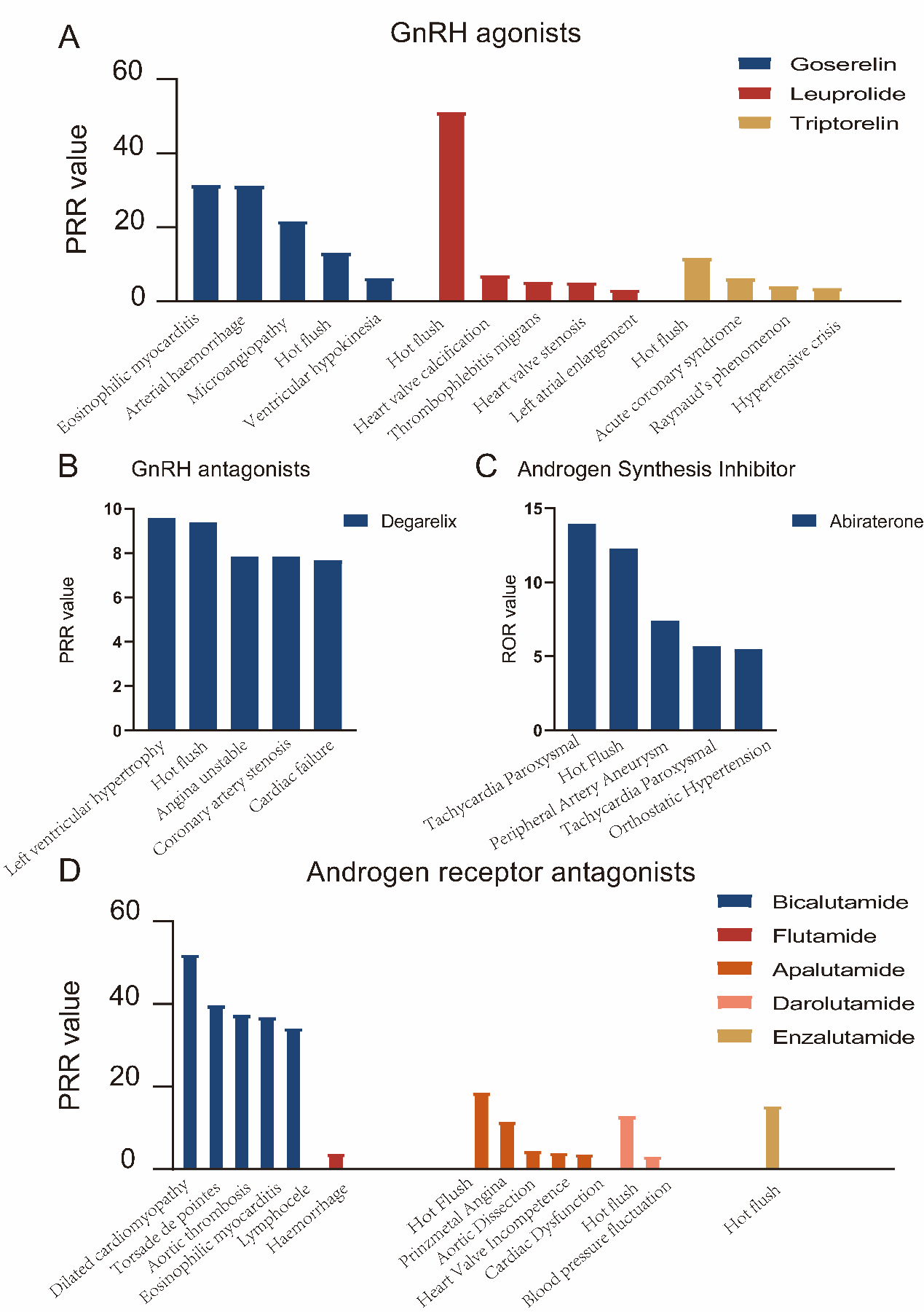
**

**Supplementary Figure 2: ADT drugs related adverse events based on disease type according to PRR value**

**
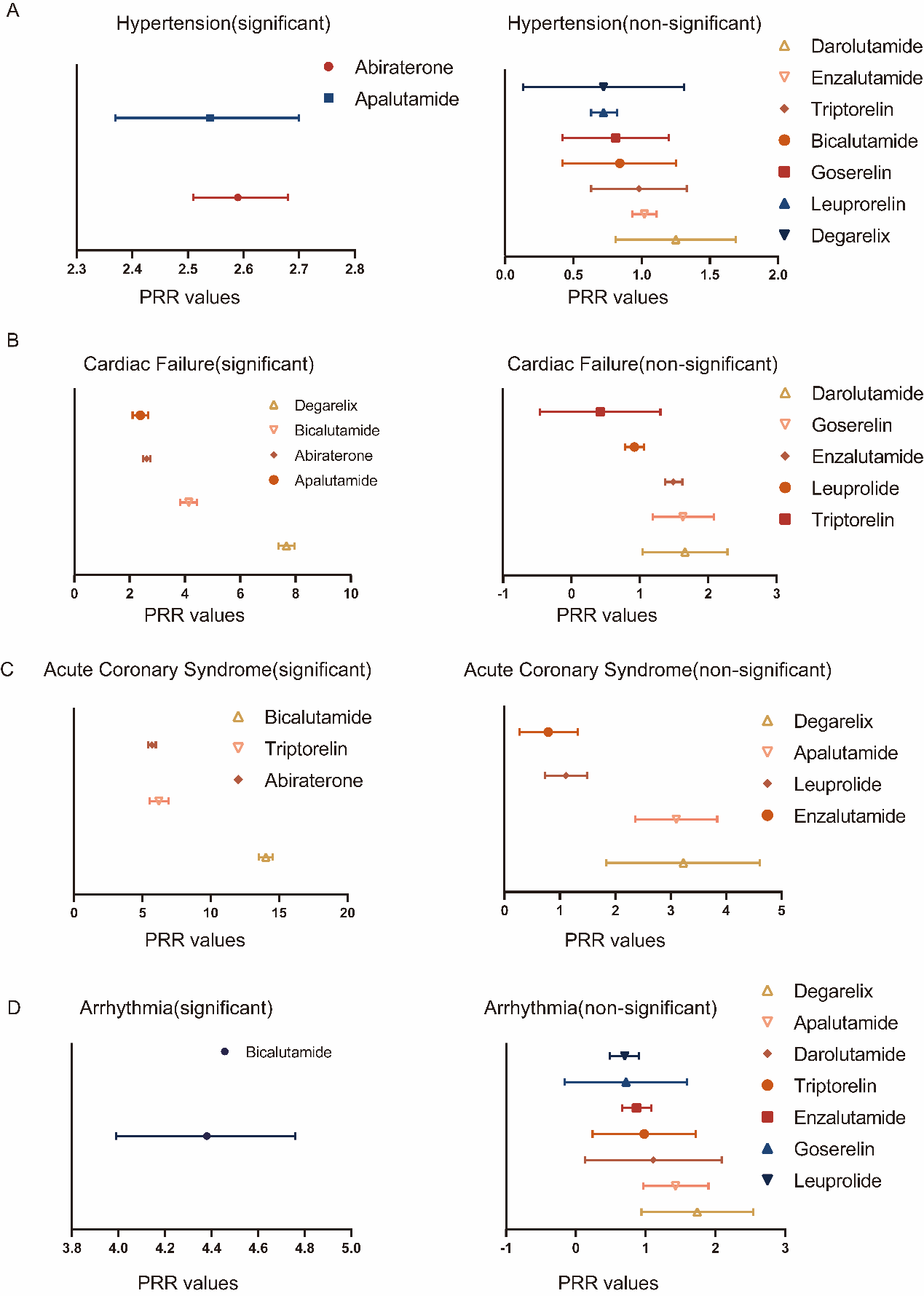
**

**Supplementary Figure 2A: ADT drugs related with Hypertension ranked by PRR value**

**Supplementary Figure 2B: ADT drugs related with Cardiac Failure ranked by PRR value**

**Supplementary Figure 2C: ADT drugs related with Cardiac Failure ranked by PRR value**

**Supplementary Table 1 : Cardiovascular adverse events of Abiraterone**

| **Adverse Effect** | **ROR [L, U]** | **PRR [L, U]** |
| --- | --- | --- |
| Tachycardia Paroxysmal | 13.96 [5.2, 37.47] | 13.96 [12.97, 14.95] |
| Hot Flush | 12.43 [11.61, 13.31] | 12.27 [12.2, 12.34] |
| Peripheral Artery Aneurysm | 7.41 [2.38, 23.09] | 7.41 [6.28, 8.55] |
| Acute Coronary Syndrome | 5.7 [4.29, 7.58] | 5.7 [5.42, 5.98] |
| Orthostatic Hypertension | 5.49 [1.76, 17.07] | 5.49 [4.35, 6.62] |
| Aortic Aneurysm Rupture | 5.07 [2.1, 12.21] | 5.07 [4.19, 5.95] |
| Torsade de Pointes | 3.69 [2.55, 5.35] | 3.69 [3.32, 4.06] |
| Hypertensive Emergency | 3.66 [1.37, 9.76] | 3.66 [2.68, 4.64] |
| Cardiotoxicity | 3.59 [2.48, 5.21] | 3.59 [3.22, 3.96] |
| Malignant Hypertension | 3.42 [1.1, 10.64] | 3.42 [2.29, 4.56] |
| Aortic Dissection | 2.63 [1.45, 4.75] | 2.63 [2.03, 3.22] |
| Aortic Stenosis | 2.63 [1.37, 5.06] | 2.63 [1.97, 3.28] |
| Cardiac Failure | 2.63 [2.29, 3.01] | 2.62 [2.48, 2.76] |
| Hypertension | 2.61 [2.39, 2.84] | 2.59 [2.51, 2.68] |
| Atrial Fibrillation | 2.6 [2.3, 2.95] | 2.59 [2.47, 2.72] |
| Hypertensive Crisis | 2.45 [1.68, 3.58] | 2.45 [2.07, 2.83] |
| Cardiac Failure Acute | 2.1 [1.22, 3.62] | 2.1 [1.55, 2.64] |

**Supplementary Table 2: Cardiovascular adverse events of Apalutamide**

| **Adverse Effect** | **ROR [L, U]** | **PRR [L, U]** |
| --- | --- | --- |
| Hot Flush | 18.96 [17.03, 21.1] | 18.56[18.46,18.67] |
| Prinzmetal Angina | 11.53 [3.71, 35.81] | 11.53[10.39,12.66] |
| Aortic Dissection | 4.46 [1.86, 10.73] | 4.46[3.59,5.34] |
| Heart Valve Incompetence | 3.97 [1.28, 12.33] | 3.97[2.84,5.1] |
| Cardiac Dysfunction | 3.62 [1.17, 11.24] | 3.62[2.49,4.75] |
| Hypertensive Crisis | 3.39 [1.83, 6.31] | 3.39[2.77,4.01] |
| Acute Coronary Syndrome | 3.1 [1.48, 6.5] | 3.1[2.36,3.84] |
| Phlebitis | 2.88 [1.08, 7.68] | 2.88[1.9,3.86] |
| Hypertension | 2.55 [2.16, 3.01] | 2.54[2.37,2.7] |
| Peripheral Coldness | 2.48 [1.29, 4.76] | 18.56[18.46,18.67] |
| Aortic Aneurysm | 2.42 [1.01, 5.83] | 11.53[10.39,12.66] |
| Cardiac Failure | 2.39 [1.81, 3.16] | 4.46[3.59,5.34] |
| Supraventricular Tachycardia | 2.37 [1.06, 5.28] | 3.97[2.84,5.1] |

**Supplementary Table 3: Cardiovascular adverse events of Bicalutamide**

| **Adverse Effect** | **ROR [L, U]** | **PRR [L, U]** |
| --- | --- | --- |
| Dilated cardiomyopathy | 51.92[16.67,161.69] | 51.9[50.77,53.04] |
| Torsade de pointes | 39.86[28.95,54.87] | 39.66[39.34,39.98] |
| Aortic thrombosis | 37.46[15.55,90.22] | 37.43[36.55,38.31] |
| Eosinophilic myocarditis | 36.85[11.85,114.61] | 36.83[35.7,37.97] |
| Lymphocele | 34.13[10.97,106.12] | 34.11[32.98,35.25] |
| Microangiopathy | 25.36[8.16,78.79] | 25.35[24.21,26.48] |
| Cardiac dysfunction | 15.33[6.88,34.17] | 15.32[14.52,16.12] |
| Acute coronary syndrome | 14.06[8.47,23.34] | 14.03[13.53,14.54] |
| Heart valve incompetence | 14.01[5.82,33.69] | 14[13.12,14.88] |
| Hot flush | 13.45[11.2,16.15] | 13.25[13.07,13.43] |
| Hypertensive crisis | 10.78[6.49,17.9] | 10.76[10.25,11.27] |
| Atrioventricular block first degree | 10.62[4.77,23.66] | 10.61[9.81,11.41] |
| Long QT syndrome | 10.34[3.33,32.11] | 10.34[9.21,11.47] |
| Atrioventricular block complete | 9.85[4.92,19.72] | 9.84[9.15,10.54] |
| Poor peripheral circulation | 9.19[3.82,22.09] | 9.18[8.31,10.06] |
| Left ventricular failure | 9.14[3.43,24.39] | 9.14[8.16,10.12] |
| Ventricular arrhythmia | 8.87[3.33,23.66] | 8.87[7.89,9.85] |
| Raynaud's phenomenon | 8.19[3.41,19.69] | 8.18[7.31,9.06] |
| Ventricular hypokinesia | 7.34[2.37,22.78] | 7.34[6.21,8.47] |
| Ventricular fibrillation | 7.18[3.86,13.35] | 7.17[6.55,7.79] |
| Bundle branch block right | 6.77[2.54,18.06] | 6.77[5.79,7.75] |
| Acute myocardial infarction | 6.1[4.05,9.18] | 6.08[5.67,6.49] |
| Ventricular extrasystoles | 6.07[3.03,12.15] | 6.07[5.37,6.76] |
| Coronary artery stenosis | 6.06[2.27,16.15] | 6.05[5.07,7.03] |
| Haemodynamic instability | 5.78[2.41,13.9] | 5.78[4.9,6.66] |
| Atrial flutter | 4.94[2.06,11.88] | 4.94[4.06,5.82] |
| Vasculitis | 4.85[2.31,10.18] | 4.85[4.11,5.59] |
| Arrhythmia | 4.39[2.99,6.45] | 4.38[3.99,4.76] |
| Cardiac failure | 4.16[3.06,5.65] | 4.14[3.83,4.44] |
| Coronary artery occlusion | 3.95[1.88,8.28] | 3.94[3.2,4.68] |
| Cardiomyopathy | 3.79[1.8,7.95] | 3.78[3.04,4.53] |
| Circulatory collapse | 3.69[1.84,7.38] | 3.69[2.99,4.38] |
| Cardiomegaly | 3.67[1.65,8.16] | 3.66[2.86,4.46] |
| Supraventricular tachycardia | 3.34[1.25,8.9] | 3.34[2.36,4.32] |
| Sinus bradycardia | 3.34[1.25,8.91] | 3.34[2.36,4.32] |
| Orthostatic hypotension | 3.21[1.53,6.75] | 3.21[2.47,3.95] |
| Atrioventricular block | 3.15[1.02,9.78] | 3.15[2.02,4.28] |
| Ventricular tachycardia | 2.93[1.32,6.53] | 2.93[2.13,3.73] |
| Angina pectoris | 2.44[1.27,4.69] | 2.44[1.78,3.09] |
| Coronary artery disease | 2.21[1.15,4.25] | 2.21[1.56,2.86] |

**Supplementary Table 4: Cardiovascular adverse events of Darolutamide**

| **Adverse Effect** | **ROR [L, U]** | **PRR [L, U]** |
| --- | --- | --- |
| Hot flush | 13.04[10.28,16.54] | 12.86[12.62,13.09] |
| Blood pressure fluctuation | 3.11[1.4,6.94] | 3.11[2.31,3.91] |

**Supplementary Table 5: Cardiovascular adverse events of Degarelix**

| **Adverse Effect** | **ROR [L, U]** | **PRR [L, U]** |
| --- | --- | --- |
| Hot flush | 9.48[7.13,12.6] | 9.38[9.1,9.66] |
| Shock | 6.38[3.43,11.86] | 6.37[5.75,6.99] |
| Pallor | 3.02[1.36,6.74] | 3.02[2.22,3.82] |
| Cardiac failure | 7.74[5.75,10.42] | 7.67[7.38,7.97] |
| Myocardial infarction | 2.74[1.98,3.81] | 2.73[2.4,3.05] |
| Angina unstable | 7.85[2.94,20.93] | 7.84[6.86,8.82] |
| Mitral valve incompetence | 3.66[1.18,11.37] | 3.66[2.53,4.79] |
| Tricuspid valve incompetence | 5.5[1.77,17.05] | 5.49[4.36,6.62] |
| Cardiomegaly | 3.17[1.02,9.82] | 3.16[2.03,4.3] |
| Left ventricular hypertrophy | 9.59[3.09,29.75] | 9.58[8.45,10.71] |
| Coronary artery stenosis | 7.85[2.53,24.35] | 7.84[6.71,8.98] |
| Cardiac failure acute | 6.61[2.13,20.5] | 6.6[5.47,7.74] |

**Supplementary Table 6: Cardiovascular adverse events of Degarelix**

| **Adverse Effect** | **ROR [L, U]** | **PRR [L, U]** |
| --- | --- | --- |
| Hot flush | 9.48[7.13,12.6] | 9.38[9.1,9.66] |
| Shock | 6.38[3.43,11.86] | 6.37[5.75,6.99] |
| Pallor | 3.02[1.36,6.74] | 3.02[2.22,3.82] |
| Cardiac failure | 7.74[5.75,10.42] | 7.67[7.38,7.97] |
| Myocardial infarction | 2.74[1.98,3.81] | 2.73[2.4,3.05] |
| Angina unstable | 7.85[2.94,20.93] | 7.84[6.86,8.82] |
| Mitral valve incompetence | 3.66[1.18,11.37] | 3.66[2.53,4.79] |
| Tricuspid valve incompetence | 5.5[1.77,17.05] | 5.49[4.36,6.62] |
| Cardiomegaly | 3.17[1.02,9.82] | 3.16[2.03,4.3] |
| Left ventricular hypertrophy | 9.59[3.09,29.75] | 9.58[8.45,10.71] |
| Coronary artery stenosis | 7.85[2.53,24.35] | 7.84[6.71,8.98] |
| Cardiac failure acute | 6.61[2.13,20.5] | 6.6[5.47,7.74] |

**Supplementary Table 7: Cardiovascular adverse events of Goserelin**

| **Adverse Effect** | **ROR [L, U]** | **PRR [L, U]** |
| --- | --- | --- |
| Eosinophilic myocarditis | 31.42[10.1,97.72] | 31.41[30.27,32.54] |
| Arterial haemorrhage | 31.26[12.98,75.28] | 31.24[30.36,32.12] |
| Microangiopathy | 21.62[6.96,67.18] | 21.61[20.48,22.75] |
| Hot flush | 13.33[11.25,15.8] | 13.14[12.97,13.31] |
| Ventricular hypokinesia | 6.26[2.02,19.42] | 6.26[5.12,7.39] |

**Supplementary Table 8: Cardiovascular adverse events of Leuprolide**

| **Adverse Effect** | **ROR [L, U]** | **PRR [L, U]** |
| --- | --- | --- |
| Hot flush | 53.9[52.68,55.15] | 51.12[51.1,51.14] |
| Heart valve calcification | 7.02[2.61,18.91] | 7.02[6.03,8.01] |
| Thrombophlebitis migrans | 5.3[1.97,14.23] | 5.3[4.31,6.28] |
| Heart valve stenosis | 5.08[2.1,12.28] | 5.08[4.19,5.96] |
| Left atrial enlargement | 3.12[1.16,8.34] | 3.12[2.13,4.1] |
| Phlebitis superficial | 3.08[1.15,8.26] | 3.08[2.1,4.07] |
| Aortic dilatation | 2.69[1.34,5.39] | 2.69[1.99,3.39] |
| Peripheral artery occlusion | 2.1[1.05,4.21] | 2.1[1.4,2.79] |

**Supplementary Table 9: Cardiovascular adverse events of Triptorelin**

| **Adverse Effect** | **ROR [L, U]** | **PRR [L, U]** |
| --- | --- | --- |
| Hot flush | 11.82[9.9,14.11] | 11.67[11.49,11.84] |
| Acute coronary syndrome | 6.22[3.11,12.44] | 6.21[5.52,6.9] |
| Raynaud's phenomenon | 4.08[1.31,12.65] | 4.08[2.95,5.21] |
| Hypertensive crisis | 3.57[1.6,7.96] | 3.57[2.77,4.37] |

**Supplementary Table 10: Cardiovascular Adverse Events related with hypertension**

| **Drug name** | **Adverse Effect** | **ROR [L, U]** | **PRR [L, U]** |
| --- | --- | --- | --- |
| Abiraterone | Orthostatic Hypertension | 5.49[1.76,17.07] | 5.49[4.35,6.62] |
|  | Hypertensive Emergency | 3.66[1.37,9.76] | 3.66[2.68,4.64] |
|  | Malignant Hypertension | 3.42[1.1,10.64] | 3.42[2.29,4.56] |
|  | Hypertensive Crisis | 2.45[1.68,3.58] | 2.45[2.07,2.83] |
| Apalutamide | Hypertensive Crisis | 3.39[1.83,6.31] | 3.39[2.77,4.01] |
| Bicalutamide | Hypertensive Crisis | 10.78[6.49,17.9] | 10.76[10.25,11.27] |
|  | Orthostatic Hypertension | 3.21[1.53,6.75] | 3.21[2.47,3.95] |
| Triptorelin | Hypertensive Crisis | 3.57[1.6,7.96] | 3.57[2.77,4.37] |
